# When Alcohol Adverts Catch the Eye: A Psychometrically Reliable Dual-Probe Measure of Attentional Bias

**DOI:** 10.3390/ijerph182413263

**Published:** 2021-12-16

**Authors:** Sera Wiechert, Ben Grafton, Colin MacLeod, Reinout W. Wiers

**Affiliations:** 1 Department of Clinical Psychology, University of Amsterdam, 1018 WT Amsterdam, The Netherlands; 2 Centre for the Advancement of Research on Emotion, School of Psychological Science, University of Western Australia, Perth 6009, Australia; ben.grafton@uwa.edu.au (B.G.); colin.macleod@uwa.edu.au (C.M.); 3 Addiction Development and Psychopathology (ADAPT)-Lab, Department of Psychology, Center for Urban Mental Health, University of Amsterdam, 1018 WT Amsterdam, The Netherlands; r.w.h.j.wiers@uva.nl

**Keywords:** attentional bias, alcohol, reliability, alcohol advertisement, dual-probe task, attentional bias modification

## Abstract

Existing tasks assessing substance-related attentional biases are characterized by low internal consistency and test–retest reliability. This study aimed to assess the psychometric properties of a novel dual-probe task to measure alcohol-related attentional bias. Undergraduate students were recruited in June 2019 (N = 63; final N = 57; mean age = 20.88, *SD* = 2.63, 67% females). In the dual-probe task, participants were presented with simultaneous visual streams of adverts promoting either alcoholic or non-alcoholic drinks, and probes were presented in both streams. The dual-probe task measured the percentage of accurately identified probes that appeared on alcohol adverts in relation to total accuracy. The dual-probe task displayed excellent split-half reliability (*M* = 0.90, *SD* = 0.11; *α* = 0.90; 95% CI [0.84, 0.93]), and the derived attentional bias measure was significantly positively associated with beer drinking in a taste-test (*r* (57) = 0.33, *p* = 0.013; 95% CI [0.07, 0.54]), with habitual drinking (*r* (57) = 0.27, *p* = 0.045; 95% CI [0.01, 0.49]), and with increased craving (*r* (57) = 0.29, *p* = 0.031; 95% CI [0.03, 0.51]). Thus, the dual-probe task assessed attentional bias with excellent internal consistency and was associated with laboratory and habitual drinking measures, demonstrating initial support for the task’s utility in addiction research.

## 1. Introduction

Cognitive biases, including attentional bias, are proposed to be factors that causally contribute to the development and maintenance of alcohol use disorders [1,2,3] (but see [4] for a critical appraisal of the current evidence). According to the attentional bias hypothesis, individual differences in alcohol consumption reflect variation in the degree to which people selectively attend to alcohol-related information [5]. This bias is thought to develop through the process of “incentive sensitization” [6,7], whereby repeated pairing of alcohol cues with alcohol use and its effects leads to the increased salience of these cues, which then capture attention.

Biased attentional responding to alcohol cues has been shown to be causally linked to drinking behavior [3,8,9,10]. However, methods of assessing attentional bias reported in prior literature have been criticized for their low psychometric reliability. Indeed, tasks commonly used to assess substance-related attentional biases (i.e., visual probe task and the modified Stroop task) typically display low internal consistency and test–retest reliability [11].

Reliable assessment of attentional bias is essential for theory testing, and previous failures to find that attentional bias and subjective craving are consistently related could reflect the poor reliability of prior attentional bias assessment tasks [8,11]. A reliable assessment of attentional bias is also needed to evaluate the impact of therapeutic procedures designed to directly attenuate maladaptive cognitive biases, collectively known as cognitive bias modification procedures (CBM) [3,12], one of which is attentional bias modification (AtBM). AtBM has shown promising results in clinical samples of patients with alcohol use disorders when delivered as an add-on to treatment [13,14,15]. However, one critical shortcoming in this line of research is that it has proven difficult to demonstrate a reliable change in attentional bias following delivery of AtBM procedures, which again may reflect the low reliability of current assessment instruments [12,16]. The capacity to reliably assess attentional bias is necessary to determine whether changes in attentional bias mediate clinical improvement. To reduce reliability problems, many studies have computed average attentional bias across multiple test sessions, but this can inflate assessment reactivity and is time- and cost ineffective [16]. Hence, recent research has sought to develop new instruments that can more reliably assess attentional bias [17]. We propose the dual-probe task to be a promising candidate [16].

Prior literature has suggested multiple reasons why the psychometric reliability of existing attentional bias measures may be poor [11,18]. These include low numbers of trials, the use of reaction time latencies, which are statistically noisy, and poor alignment between stimuli and sample behavior (e.g., type of alcohol preferred may not align with the alcoholic beverage presented in the task) [11,18]. The present implementation of the dual-probe task has been developed to reduce these problems, hereby representing an improved version of the visual probe task [16,19]. Specifically, in this new task, we delivered three blocks of trials to increase the total number of trials (i.e., 60 trials more than the number of 220 trials suggested by Ataya et al.) [11]. Second, left and right video streams were presented simultaneously, with audio played to the corresponding ear. One stream presented alcohol-related information, and the other presented non-alcohol-related information. These streams switched their location once every few seconds. In contrast to the original visual probe task, at semi-random intervals, two different probes were simultaneously presented very briefly (200 ms), one in the location of alcohol-related information and the other in the location of non-alcohol-related information (vs. only one probe in either location). Participants identified whatever probes they saw. Rather than measuring average reaction time to identify single probes in the location of alcohol vs. non-alcohol-related information (as in the original visual probe task), the proportion of probes participants correctly identified in the location of alcohol-related information provided an index of attentional bias to such information. In this way, reaction time measurement had no value in determining attentional bias in the current task. An attentional bias to the one condition (e.g., alcohol) in the present study would not result in a differential reaction time to identify probes but rather is only calculated by the number of identifications on one location compared to the other. Therefore, we argue to have decreased measurement noise by employing a more direct measure of the attention that is independent of reaction times. Third, we paid particular attention to recruiting participants who enjoyed beer, and the task contained beer adverts.

When determining which visual streams to use in our alcohol dual-probe task, we chose adverts for alcoholic and non-alcoholic drinks, as they are developed to attract and capture attention. Alcohol adverts have been found to induce subjective craving [20,21], and are often designed to increase young people’s desire to consume alcohol [22]. They have been shown to act as conditioned stimuli, thereby increasing subjective craving, motivation to drink, and actual drinking behavior [21,22,23,24,25]. Alcohol adverts may influence the individual on an intrapersonal level, but they can also affect the formation of the individual’s social norms, with the potential to create a drinker’s identity and promote greater acceptance of alcohol use [26].

Some prior research studies have examined the relationship between attentional bias to alcohol-related information and drinking behavior. For example, an eye-tracking study by McAteer et al. (2015) found that adolescent drinking was associated with the time spent fixating on alcohol stimuli [27]. Further, Field et al. (2004) demonstrated that heavy social drinkers, compared with light social drinkers, exhibited heightened attentional bias towards alcohol pictures, which also correlated with subjective alcohol craving [20]. Morgenstern et al. (2014) showed that susceptibility to alcohol adverts was related to binge drinking [28], while neuroimaging has shown that the degree to which viewing alcohol adverts elicits activation in the reward system correlates with self-reported drinking [29].

Although exposure to alcohol adverts is ubiquitous, people vary in their consumption, permitting the possibility that differential attention to alcohol adverts may impact consumption behavior. Inherently, alcohol adverts are constructed to induce craving [22], and attentional bias has been shown to predict levels of subjective craving in response to alcohol-related stimuli [8]. Therefore, we argue that alcohol adverts may reflect accurate stimuli to measure attentional bias in the dual-probe task.

For this, the primary goal of the current study was to validate the dual-probe task using advertisement stimuli in which the internal consistency (i.e., split-half reliability) of the task was investigated. In addition to this primary goal, associations between the attentional bias score yielded by the dual-probe task, laboratory-based measures of drinking (i.e., subjective craving, beer consumption in a sham taste test), habitual alcohol use, and attentional control were explored.

## 2. Materials and Methods

### 2.1. Participants

Undergraduate students from the University of Amsterdam were recruited for research credits or monetary compensation (1.5 credits or EUR 15, respectively). The following inclusion criteria applied: minimum age of 18 (legal drinking age), beer drinkers, negative blood alcohol content at testing (i.e., as acute intoxication can alter attentional bias and craving) [30], and no ongoing self-reported psychological treatment (to ensure that alcohol consumption in the study could not interfere with such treatment). Prior to participation, participants were informed that the goal of the study was to investigate how individuals perceive advertisement videos, framed as a consumer satisfaction study, containing a taste test of alcoholic and non-alcoholic drinks. Participation was entirely voluntary; thus, participants were free to withdraw at any time, without any consequences. The length of the experimental session was 90 min. The study protocol was reviewed and approved by the Ethics Committee of the University of Amsterdam (reference: 2019-DP-10370; date of approval: 1 April 2019) and was conducted in accordance with the Declaration of Helsinki.

### 2.2. Materials

#### 2.2.1. Questionnaires

Participants’ demographic information was obtained (i.e., gender (1 = male; 2 = female; 3 = other); age (continuous variable); ethnicity (1 = Caucasian; 2 = Hispanic; 3 = African American; 4 = Asian; 5 = Other)). Further, the following battery of questionnaires was administered (full data on https://osf.io/jrfx6/, accessed on 15 December 2021): the Alcohol Use Disorders Identification Test (AUDIT) [31]; a self-report timeline follow-up procedure (i.e., for the past 14 days) [32]; attentional control [33]; other substance use; 11-point Likert scales for craving and thirst. For the study, changes in subjective craving for beer and habitual alcohol use measures were included. To assess the change in subjective craving (from before to after the dual-probe task), we computed a change score between two time points (i.e., before the task subtracted from after the task), obtained from an 11-point Likert scale assessing craving for beer: “I want to drink beer right now” (1—not at all, 11—extremely). This assessment was delivered three times within the experimental session (before (T1) and after (T2) the dual-probe task, as well as after the taste test (T3)) of which we used the first two measurements for the analysis at hand (T2 subtracted by T1). A higher craving change score reflected increased subjective craving after the dual-probe task, relative to before exposure. To create a measure of habitual alcohol use, the AUDIT-C measure (i.e., first three questions of AUDIT) [34] and the total of the timeline follow-up measure were scaled and averaged (their correlation in our sample was *r* (57) = 0.63, *p* < 0.001, 95% CI [0.44, 0.76]). A higher score on this measure indicated greater habitual use of alcohol.

#### 2.2.2. Video Advertisement Stimuli

**Figure 1:**
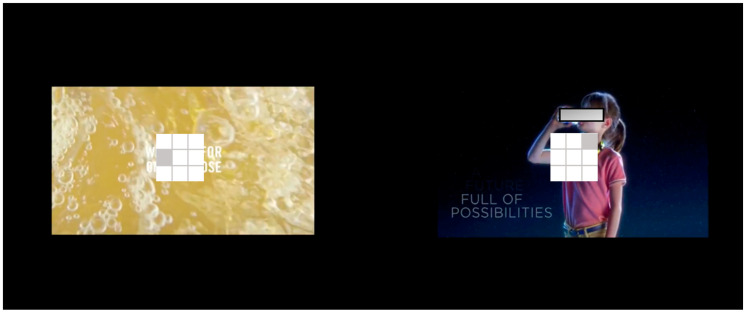
An example figure of the experimental stimuli used in the dual-probe task, with a beer advert on the left, and a water advert on the right side of the example. The left probe stimulus corresponds to a “4” response on the number pad. The right probe stimulus corresponds to a “9” response on the number pad.

Dual videos comprising alcohol and non-alcohol-related advertising were created for presentation within the dual-probe attentional assessment task (see Figure 1 for a simplified example). These dual videos were constructed using 16 beer adverts (8 distinct beer brands, with 2 different adverts per brand) and 16 soft-drink adverts (8 distinct soft-drink brands, with 2 different adverts per brand). In each dual video, one stream displayed the 16 alcohol adverts, and the other stream displayed the 16 soft-drink adverts. At any point in time, the advert from one stream was playing in the center of the right half of the screen, while the advert from the other stream was playing in the center of the left half of the screen. The audio track for each advert played through the audio channel corresponding to the side of the screen on which that advert appeared. Thus, participants were always simultaneously exposed to an advert for a beer and an advert for a soft drink. At semi-random intervals (constrained to be between 5 and 7 s), the positions of the two advert streams switched places, with both the visual and audio content of the stream previously being presented on the left now switching to the right and vice versa. The use of competing streams of audio has been a long-standing approach adopted in research investigating individual differences in selective attention [35]. In such research, it has repeatedly been demonstrated that people can fully comprehend information communicated within the audio stream to which they are attending, while successfully ignoring information communicated within the unattended audio stream [35], justifying our decision to adopt this approach.

In the current study, three dual videos were presented across three blocks. Each dual video presented the same adverts, but these were delivered in different orders and different pairings across blocks. Each dual video lasted a total of 555 secs, and within each dual video, the positions of the two advert streams were switched 94 times.

#### 2.2.3. Dual-Probe Attention Assessment Task

This task was designed to measure the degree to which participants selectively attended to the alcohol adverts, compared with the soft-drink adverts. The task was programmed in BBC Basic (for Windows 5.94a). In a separated laboratory cubicle of the University of Amsterdam, participants were seated in front of a 24-inch desk computer (i.e., head and screen on the same height) at a 50 cm distance to the screen and used the separated number pad of the keyboard to give responses in the dual-probe task.

Across three blocks, participants viewed dual videos on a black background while wearing stereo headphones. While they were allowed to view the videos as they pleased, they were instructed to identify probe stimuli whenever they saw these briefly appear on the screen. During the presentation of the dual videos, probe pairs were presented, for 200 ms, at pseudorandom intervals. One member of each probe pair appeared in the center of the left video, and the other member appeared in the center of the right video. These probes were each grey, 3 × 3 grids, in which one of the outer eight positions in any such grid was shaded by a small grey square. Participants had to correctly identify the shaded grid position in any probe they saw, by quickly pressing the corresponding key on their 3 × 3 number pad (see Figure 1 for an example). In this task, a response was coded as a correct probe identification when the first button press made within 2000 ms of a probe pair’s appearance matched the identity of one of the two probes in that pair. When the first button press made within 2000 ms of a probe pair’s appearance did not match any of the two probes, or when no button press was made within 2000 ms of a probe pair’s appearance, it was recorded that no probe was correctly identified from that pair. Reaction time data were only collected to ensure that a correct response was defined in terms of pressing the correct button in the 2000 ms window following the probe pair’s appearance. Though participants were told that the identity of the two probes in each briefly presented pair was the same, the identities of the probes in each pair were always different from each other. We informed participants that the probe identities were equivalent to discouraging active efforts of participants to apprehend the identity of both probes in the presented pairs. Rather, participants simply responded to the probe they saw, which we assumed would be the one appearing at the attended location. Hence, the probe that participants correctly identified revealed which of the two adverts they were attending to when the probe pairs were presented. A percentage score indexing attentional bias to alcohol adverts relative to soft-drink adverts was computed for each participant. This was calculated as follows: the number of correctly identified probes in the location of the alcohol adverts divided by the total number of correctly identified probes (range: 0–100, with higher numbers indicating greater attention to alcohol adverts). Attentional bias to alcohol adverts can be indexed only if participants follow the instruction to correctly identify probes that they see. As such, we set our low probe accuracy participant exclusion criterion as failure to correctly react to and identify a probe on at least 75% of the probe pair presentations.

#### 2.2.4. In Vivo Measure of Alcohol Consumption: Sham Taste Test

To objectively assess differences in alcohol consumption within the controlled experimental setting, participants were presented with 200 mL of four beverages (two brands of beer and two brands of cola) and were informed that their goal was to, by taste alone, guess the beverages’ product brands [36]. Participants were given 10 min to complete this task. Unbeknown to participants, the measure of interest was the amount of beer they consumed during the 10 min period, expressed in ml. This was computed by weighing the amount of beer remaining after consumption and subtracting this from the original content of the cup. Thus, higher scores represented greater amounts of beer consumed.

### 2.3. Procedure

Each participant individually completed a single test session in a laboratory cubicle, which occurred only if informed consent was given. Participants first completed the questionnaire battery, followed by the dual-probe task. Participants were instructed to view the videos as they pleased and to identify whatever probe stimuli they saw appear on the screen. In the dual-probe task, participants completed three blocks that each lasted 555 s with a total duration of approximately 30 min. Across the three blocks, participants were shown 282 probe pairs (94 probe pairs in each block). After the dual-probe task, the taste test was conducted. As stated above, subjective craving for beer was assessed at three time points: before and after exposure to the adverts in the dual-probe task, as well as after the taste test. The duration of the test session was approximately 90 min. Following the test session, participants were debriefed and thanked for their participation.

### 2.4. Statistical Analysis

Analyses were conducted in RStudio (Version 1.2.5019). First, the internal reliability of the attentional bias measure was determined using a split-half analysis conducted with the multicon [37] and splithalfr packages [38]. Following this, bivariate Pearson and Spearman correlations between attentional bias (as provided by the dual-probe task) and demographics of interest (gender, age), habitual alcohol use, attentional control, and laboratory measures of drinking (change in subjective craving, beer consumption in the sham taste test) were examined. As an additional analysis, two hierarchical linear regressions were conducted on the effect of the attentional bias score on laboratory measures of drinking (change in subjective craving; beer consumption in the sham taste test; controlling for gender and age in step 1; habitual use and attentional control added as covariates in step 2).

## 3. Results

### 3.1. Sample Characteristics

In the initial sample of 63 participants, 1 participant was excluded due to a positive breath alcohol test at the beginning of the experiment, 4 participants were excluded as they responded to less than 75% of the probes, and 1 participant was excluded as they correctly identified a probe on less than 75% of trials on which they responded to a probe. Thus, the final analytical sample consisted of 57 participants (38 female; 47 Caucasian, see Table 1 for an overview of ethnicity distributions), with a mean age of 20.88 (*SD* = 2.63). Accuracy on the responded trials in the dual-probe task was high in this final sample, with a mean of 94.77% (*SD* = 4.61). The mean attentional bias to alcohol index score in the sample was 48.97 (*SD* = 9.40), and the mean reported change in subjective craving for beer was 1.02 (*SD* = 1.51).

### 3.2. Internal Reliability of the Attentional Bias to Alcohol Adverts

Split-half reliability analysis was conducted on the attentional bias to alcohol adverts index scores. The mean of the split-half correlations was 0.82, and the mean of the split-half reliabilities was 0.90 (*SD* = 0.11), with a Cronbach’s alpha of 0.90. The bootstrapped lower and upper bound of the mean internal consistency measure was 0.84 and 0.93, respectively.

### 3.3. Association between Attentional Bias to Alcohol Adverts and Drinking Measures

Correlational analyses revealed that greater attentional bias to alcohol adverts was significantly associated with increased subjective craving after the task (*r* (57) = 0.29, *p* = 0.031; 95% CI [0.03, 0.51]), greater amounts of beer consumed in the taste test (*r* (57) = 0.33, *p* = 0.013; 95% CI [0.07, 0.54]), and more habitual use of alcohol (*r* (57) = 0.27, *p* = 0.045; 95% CI [0.01, 0.49]). Attentional bias was not significantly associated with attentional control (*r* (57) = −0.06, *p* = 0.644; 95% CI [−0.32, 0.20]; see Table 2).

Using hierarchical regression, we proceeded to examine whether attentional bias to alcohol by the dual-probe task continued to predict change in subjective craving to beer and to predict the amount of beer consumed in the taste test while controlling for demographics (i.e., gender, age), habitual alcohol use, and attentional control.

We first conducted a hierarchical regression in which change in subjective craving to beer scores was the dependent variable. In the regression model containing gender, age and the attentional bias to alcohol scores as predictor variables (i.e., step 1), attentional bias to alcohol significantly predicted independent variance in the change in subjective craving to beer scores (*β* = 0.28, *p* = 0.036). However, when attentional control and habitual alcohol use were added into the model as predictor variables (i.e., step 2), this was no longer the case (see Table 3).

Next, we conducted a hierarchical regression in which beer consumption in the taste test was the dependent variable. In the regression model containing gender, age and the attentional bias to alcohol scores as predictor variables (i.e., step 1), attentional bias to alcohol significantly predicted independent variance in the amount of beer consumed in the taste test (*β* = 0.29, *p* = 0.020). However, when attentional control and habitual alcohol use were added into the model as predictor variables (i.e., step 2), the attentional bias score no longer predicted independent variance in the amount of beer consumed (*β* = 0.20, *p* = 0.094; see Table 4).

## 4. Discussion

As previous instruments assessing attentional bias were shown to have insufficient, if not poor, reliability [8,11], the current study developed a new variant of the dual-probe approach, in which attentional bias to alcohol adverts was assessed. The resulting dual-probe measure of attentional bias showed high internal consistency. Additionally, attentional bias to alcohol adverts was positively associated with habitual drinking, beer consumption in the taste test, and an increase in subjective craving for beer following exposure to the adverts in the dual-probe task, underscoring its predictive validity. When controlling for demographics, attentional bias continued to predict laboratory measures of alcohol use, but these associations became non-significant when adding real-life self-report measures related to alcohol use. Nevertheless, we argue that these analyses are still promising. Attentional bias, as measured by the dual-probe task, is an objective measure that was still directly correlated with real-life and laboratory measures of drinking. In contrast to self-report measures, the dual-probe task can be experimentally manipulated and adapted in the future as an AtBM training tool to directly investigate the resulting behavioral change.

To our knowledge, this is the first study to have validated a dual-probe approach to assess attentional bias when using alcohol adverts as experimental stimuli and to have demonstrated that this attentional bias measure had good internal consistency. Adverts represent a communicative tool intended to convince an audience to purchase or take action toward the product shown [39], specifically by increasing subjective craving [20,21]. In line with this, we demonstrated that, as much as the alcohol adverts used in our dual-probe task captured attention, so too did craving increase, and this attention bias measure correlated with laboratory and real-life measures of drinking.

We underline the fact that there was no association between attentional bias to alcohol adverts and attentional control in the present study. This may have been because participants in the dual-probe task were instructed to let their attention flow and switch as they pleased. Hence, attentional control may have been unrelated to the attentional bias, as participants were not directed to make use of attentional control. Additionally, recent studies suggest that self-reported attentional control may more strongly reflect people’s beliefs about their attentional control rather than their actual attentional control ability [40]. In the present study, we employed a self-report measure of attentional control, and so the absence of an association between attentional bias and attentional control may reflect the fact that participants’ genuine ability to control their attention was not assessed. Thus, we suggest that future researchers who wish to investigate the association between the patterns of attentional selectivity revealed by the current dual-probe approach and attentional control should employ performance-based procedures for assessing attentional control or attentional control training tasks such as the dual n-back task [41,42]. In this way, the association between attentional bias and attentional control could be, respectively, addressed by investigating the naturally occurring association between these two variables, or by determining whether the direct modification of attentional control serves to alter the patterns of attentional selectivity revealed by the dual-probe task.

### 4.1. Limitations

First, we recognize that the relatively small sample size constraints power, and additionally, that the study procedures were not pre-registered. Nevertheless, a sensitivity power analysis based on one-tailed correlations indicates that the design was adequately powered to detect moderate-to-large correlations (i.e., 0.32). Second, the current study concerned only beer drinkers, exclusively using beer adverts as alcohol-related stimuli, and beer beverages in the sham taste test. It remains to be seen whether the observed effects will be replicated using other types of alcohol adverts, and with taste tests using different types of alcoholic beverages. Third, prior to consenting to participation, candidate participants were informed that the study procedure included an alcoholic taste test. We acknowledge that this may have led to selection bias. Specifically, participants who enjoy drinking alcohol may have been more prone to participate. Therefore, future studies should aim to recruit participants from diverse drinking groups (i.e., light, moderate, and heavy drinkers) to counter the possibility of such selection bias. Fourth, the study used only a single predominantly Caucasian student population (sensitivity analyses on a Caucasian subsample revealed broadly the same conclusions, i.e., excellent internal consistency, significant associations with laboratory measures of alcohol use. We found reductions in the associative strength between the dual-probe attentional bias score and self-reported habitual alcohol use. We emphasize that differences in results may be related to smaller sample size and, thereby, reduced power). Such a sample is restricted in terms of age, ethnicity, educational status, and other potential participant characteristics. Finally, although our findings confirm the internal consistency of our attentional bias measure, we cannot comment on whether it also satisfies the need recognized to develop attentional bias measures that have good test–retest reliability [8,11], which should now be the focus of future research.

### 4.2. Implications

Despite these limitations, this initial validation of the dual-probe task to assess attentional bias to alcohol-related information was undertaken based on a strong theoretical and methodological rationale and showed encouraging initial value as an assessment approach. Participants demonstrated good accuracy in identifying probes, and the resulting attentional bias measure had high internal consistency and predicted drinking-related variables. We suggest that this exploratory study should be replicated in future pre-registered studies that employ larger and more diverse samples, to confirm the generality of the results with a higher power. Such future extensions further map out the reliability and validity of the task, using designs that allow determination of test–retest reliability, as well as the test-predicted relationships between the attentional bias scores yielded by this dual-probe task and additional self-report or drinking measures.

Though prior research indicates that attentional bias to alcohol-related information may play an important role in alcohol use disorders [1,2,3,5], testing this has been compromised by the low reliability of previous attentional bias measures. Indeed, prior inconsistencies across research, concerning evidence of an association between attentional bias to alcohol-related information and alcohol use, may reflect the low reliability of these prior attentional bias assessment tasks, rather than an absence of an association between such attentional bias and alcohol use [43,44]. Therefore, there is a strong need for new assessment methods that reliably measure this attentional bias [17]. The current dual-probe assessment approach has demonstrated good internal consistency and predictive validity, and therefore, we hope it will prove to be of value to fellow researchers.

We also hope that after sufficient validation, variants of this dual-probe approach may successfully train reduced attentional bias to alcohol adverts in individuals with alcohol problems or use disorders. Implemented as a training tool for AtBM, such task variants could serve as potential add-ons to existing treatments, ultimately reducing individuals’ alcohol consumption over time, while permitting the assessment of the hypothetical mediating role of a change in attentional bias with a more reliable instrument [12,13,14,15].

## 5. Conclusions

In this exploratory study, designed to validate the dual-probe approach using alcohol adverts, the attentional bias score displayed excellent internal consistency and significantly predicted habitual alcohol use and laboratory measures of drinking. We greatly hope this research inspires further investigation, and that future preregistered studies delivering this assessment approach to larger and more diverse samples will further advance understanding in ways that enable theoretical progress while also delivering real-world applied benefits.

## Figures and Tables

**Table 1:** Percentages of ethnicity groups in the current sample.

Ethnicity Group	Count (Percentage)
Caucasian	47 (82.46%)
Hispanic	1 (1.75%)
African American	0 (0)
Asian	4 (7.02%)
Hindustani	1 (1.75%)
Multiple groups reported	4 (7.02%)

Total N = 57 participants.

**Table 2:** Means, standard deviations, and correlations with confidence intervals between variables of interest.

Variable	*M*	*SD*	1	2	3	4	5	6
1. Gender	1.67	0.48						
2. Age	20.88	2.63	−0.00 (0.09)					
			[−0.27, 0.26]					
3. Habitual Use	0.00	0.90	−0.19 (−0.26 *)	0.14				
			[−0.43, 0.07]	[−0.12, 0.39]				
4. Dual-Probe Score	48.97	9.40	−0.11 (−0.01)	0.02	0.27 *			
			[−0.36, 0.16]	[−0.24, 0.28]	[0.01, 0.49]			
5.Subjective Craving	1.02	1.51	−0.04 (−0.04)	0.16	0.13	0.29 *		
			[−0.30, 0.22]	[−0.10, 0.41]	[−0.14, 0.38]	[0.03, 0.51]		
6. Beer Consumption	81.16	60.98	−0.36 ** (−0.36 **)	0.16	0.37 **	0.33 *	0.16	
			[−0.57, −0.11]	[−0.11, 0.40]	[0.12, 0.58]	[0.07, 0.54]	[−0.11, 0.40]	
7. Attentional Control	52.49	8.06	−0.13 (−0.12)	−0.07	0.12	−0.06	−0.36 **	−0.22
			[−0.38, 0.14]	[−0.32, 0.20]	[−0.15, 0.37]	[−0.32, 0.20]	[−0.57, −0.11]	[−0.45, 0.04]

*M* and *SD* are used to represent mean and standard deviation, respectively. Values in square brackets indicate the 95% confidence interval for each correlation. For the variable gender, Spearman’s correlation 𝜌 is presented in brackets. N = 57 participants. * *p* < 0.05. ** *p* < 0.01.

**Table 3:** Gender, age, dual-probe score, habitual use, and attentional control regressed on change in subjective craving.

Variable	Estimate	SE	95% CI		*β*	*p*
			*LL*	*UL*		
**Step 1**				*p* = 0.110, Adjusted R^2^ = 0.06 F = 2.11, df = 3, 53		
Gender	−0.03	0.41	−0.86	0.80	−0.01	0.939
Age	0.09	0.07	−0.06	0.24	0.16	0.229
Dual-Probe Score	0.05	0.02	<0.01	0.09	0.28	0.036
**Step 2**				*p* = 0.020, Adjusted R^2^ = 0.15 F = 2.96, df = 5, 51		
Gender	−0.14	0.40	−0.95	0.66	−0.05	0.723
Age	0.07	0.07	−0.07	0.21	0.12	0.330
Dual-Probe Score	0.04	0.02	-<0.01	0.08	0.24	0.074
Habitual Use	0.13	0.22	−0.31	0.58	0.08	0.547
Attentional Control	−0.07	0.02	−0.11	−0.02	−0.35	0.008

95% confidence interval (CI) of the estimate; SE = standard error; *β* = standardized beta coefficient.

**Table 4:** Gender, age, dual-probe score, habitual use, and attentional control regressed on beer consumption.

Variable	Estimate	SE	95% CI		*β*	*p*
			*LL*	*UL*		
**Step 1**				*p* = 0.002, Adjusted R^2^ = 0.19 F = 5.48, df = 3, 53		
Gender	−42.20	15.48	−73.24	−11.15	−0.33	0.009
Age	3.45	2.79	−2.14	9.04	0.15	0.222
Dual-Probe Score	1.88	0.78	0.31	3.45	0.29	0.020
**Step 2**				*p* < 0.001, Adjusted R^2^ = 0.30 F = 5.72, df = 5, 51		
Gender	−41.25	14.77	−70.89	−11.60	−0.32	0.007
Age	2.12	2.64	−3.18	7.42	0.09	0.426
Dual-Probe Score	1.30	0.76	−0.23	2.82	0.20	0.094
Habitual Use	18.73	8.13	2.40	35.07	0.28	0.025
Attentional Control	−2.08	0.87	−3.82	−0.34	−0.27	0.020

95% confidence interval (CI) of the estimate; SE = standard error; *β* = standardized beta coefficient.

## Data Availability

The data presented in this study are openly available in OSF at https://osf.io/jrfx6/ (accessed on 15 December 2021). Additionally, a copy of the task is available upon request.
